# Development of a CT-Based Auto-Segmentation Model for Prostate-Specific Membrane Antigen (PSMA) Positron Emission Tomography-Delineated Tubarial Glands

**DOI:** 10.7759/cureus.31060

**Published:** 2022-11-03

**Authors:** Caleb Sample, Naomi Jung, Arman Rahmim, Carlos Uribe, Haley Clark

**Affiliations:** 1 Physics and Astronomy, University of British Columbia, Vancouver, CAN; 2 Medical Physics, BC Cancer Surrey, Surrey, CAN; 3 School of Biomedical Engineering, University of British Columbia, Vancouver, CAN; 4 Physics and Astronomy/Radiology, University of British Columbia, Vancouver, CAN; 5 Integrative Oncology, BC Cancer Research Institute, Vancouver, CAN; 6 Radiology, University of British Columbia, Vancouver, CAN; 7 Functional Imaging, BC Cancer Vancouver, Vancouver, CAN; 8 Physics and Astronomy/Medicine, University of British Columbia, Vancouver, CAN

**Keywords:** psma, optimization, xerostomia, radiotherapy, autosegmentation, saliva glands, psma pet, tubarial

## Abstract

Modern inverse planning radiotherapy techniques allow for organs at risk (OARs) to evade radiation doses that they would have been subjected to with earlier techniques. The extent to which patient outcomes may be improved using these techniques depends on the delineation accuracy of target volumes and OARs on medical images as well as clinical dose constraints applied to regions of interest (ROIs). The recent discovery of bilateral “tubarial” salivary glands, which were found in the nasopharynx using prostate-specific membrane antigen (PSMA) positron emission tomography (PET), raises concerns over how dose to this region might affect patient outcomes. The dose response of the major salivary glands is known to be variable, and it is possible that the dose in tubarial glands constitutes a missing variable in the optimization of head and neck (HN) radiotherapy plans. A necessary first step toward conducting clinical studies that include the tubarial glands in plan optimization is to develop methods for delineating these glands without the use of PSMA PET images, as their acquisition is not considered a part of the standard of care for HN patients. In this study, we develop an open-source program, Organogenesis, for the auto-segmentation of tubarial glands using only computed tomography (CT) images. A predictive model is trained using contours derived from PSMA PET images, allowing for accurate delineation of tubarial glands, which cannot be manually contoured using CT only. Organogenesis provides a predictive model for tubarial glands that can be iteratively improved on with additional data, creating a viable pathway to clinical studies that can assess the importance of incorporating tubarial glands into HN radiotherapy plan optimization.

## Introduction

A challenging aspect of creating high-quality radiotherapy treatment plans is providing tumoricidal doses to target volumes while simultaneously sparing normal tissue. This is a multi-criteria optimization problem that requires oncologists to make trade-offs between tumor dose coverage and normal tissue irradiation. This is especially true during the creation of treatment plans for head and neck (HN) cancer patients, where salivary glands often abut or overlap with target volumes [1]. It is common for saliva production in post-therapy HN patients to be severely impaired [2] as a result of salivary gland irradiation. This results in a subjective condition called xerostomia, or self-reported “dry mouth.” Xerostomia and hyposalivation are known to severely impact the quality of life by hindering or eliminating the ability to speak, chew, taste, or swallow [3] and may cause oral infections, dental caries, and other oral sequelae [4-6]. The three major salivary glands include the parotid, submandibular, and sublingual glands. Irradiation of parotid glands has been found to be the greatest risk factor for xerostomia following treatment [7].

To achieve satisfactory organ sparing during radiotherapy, treatment planning workflows must include methods for defining the patient’s geometry and anatomy via medical imaging and image segmentation. Typically, computed tomography (CT) is used to acquire images, which are then used for the delineation of organs at risk (OARs).

Advances in machine learning techniques have made possible automatic segmentation (auto-segmentation) of objects in images, most commonly using convolutional neural networks (CNNs). The quality of auto-segmentation algorithms significantly improved with the development of the U-Net architecture [8] in 2015, which allowed for clinical adoption of auto-segmentation software for delineating regions of interest (ROIs) to become a viable reality. U-Net is a CNN architecture divided into a contraction path for extracting features (encoder) and an expansion path for constructing mask images using these extracted features (decoder). The encoder iteratively reduces the image size and increases the number of channels by means of a series of double 3 x 3 convolutions with numerous kernels, and intermittent activation functions such as rectified linear units (ReLU) [9] as well as max-pooling operations. A symmetric decoder then up-samples images back to their original size while reducing the number of channels using a series of transposed convolutions, 3 x 3 convolutions, and activation functions. Lastly, in what are called skip connections, the output of each activation function within the encoder is concatenated with the output of a transposed convolution in the decoder at a symmetric level within the pipeline.

In modern times, it is perhaps taken for granted that the anatomy of the human body has been scrupulously studied to a level where all relevant OARs are understood and considered during radiotherapy plan optimization. However, an unaccounted-for pair of bilateral salivary glands in the posterior nasopharynx region was discovered in early 2021 [10] and has been named the tubarial glands. This discovery was made by observing full-body prostate-specific membrane antigen (PSMA) positron emission tomography (PET) and CT images. PSMA PET involves the injection of a radiotracer, which binds to the PSMA, a transmembrane protein specific to prostate cells including carcinoma. This radiotracer is also found to have an affinity for salivary and lacrimal gland tissue, rendering PSMA PET a viable method of delineating salivary glands in addition to the prostate. While viewing full-body PSMA PET images, it was noticed [10] that radiotracer uptake in the posterior nasopharynx region resembled uptake observed in major salivary glands. The finding was confirmed by observing PSMA PET images for 100 consecutively scanned patients and with a human cadaver study. The average length of the glands was found to be approximately 4 cm. The same group also retrospectively assessed the correlation between tubarial gland mean dose during radiotherapy and post-treatment xerostomia after 12 months, finding a significant correlation (p = 0.007). It is unknown whether a high tubarial gland dose exacerbates symptoms of xerostomia but this correlation suggests that including the tubarial glands in radiotherapy plan optimization is worth considering, and could potentially improve HN patient outcomes.

The existence of the tubarial glands as major salivary glands is not without debate [11-17], and this contention would be best settled through experimentation, by testing the impact of treating tubarial glands as OARs during radiotherapy plan optimization. A logical next step in assessing the impact of the dose on these glands is to carry out a clinical study, comparing patient outcomes for plans created with and without tubarial gland dose constraints. However, segmentation of the tubarial glands currently requires PSMA PET, which is costly and not routinely acquired for HN patients.

The aim of this work is to develop an auto-segmentation model for the tubarial glands, which requires only CT images. This will allow for streamlined delineation of the glands without requiring PSMA PET images or changes to existing clinical workflows. Having the ability to contour tubarial glands with only CT images will allow further research into the role of tubarial glands in radiotherapy.

## Materials and methods

Manual delineation of PSMA PET images

Registered (18F) DCFPyL PSMA PET and radiotherapy treatment planning CT images for a small cohort of prostate cancer patients (n = 30, age: 68 ± 13, weight: 90kg ± 38kg) having previously received treatment were collected for model training. DICOMautomaton [18] was used for manual segmentation and Digital Imaging and Communications in Medicine (DICOM) file export of tubarial gland structures using PSMA PET images. As absolute PSMA PET uptake varies from patient to patient, images were normalized to standard uptake values based on body weight (SUVbw). A lower threshold of SUVbw = 1 was applied to PET images to estimate the location of tubarial glands borders. The right and left tubarial glands were then manually delineated slice by slice around the threshold-defined perimeters and the resulting structures were exported as DICOM files.

Pre-processing

**Figure 1:**
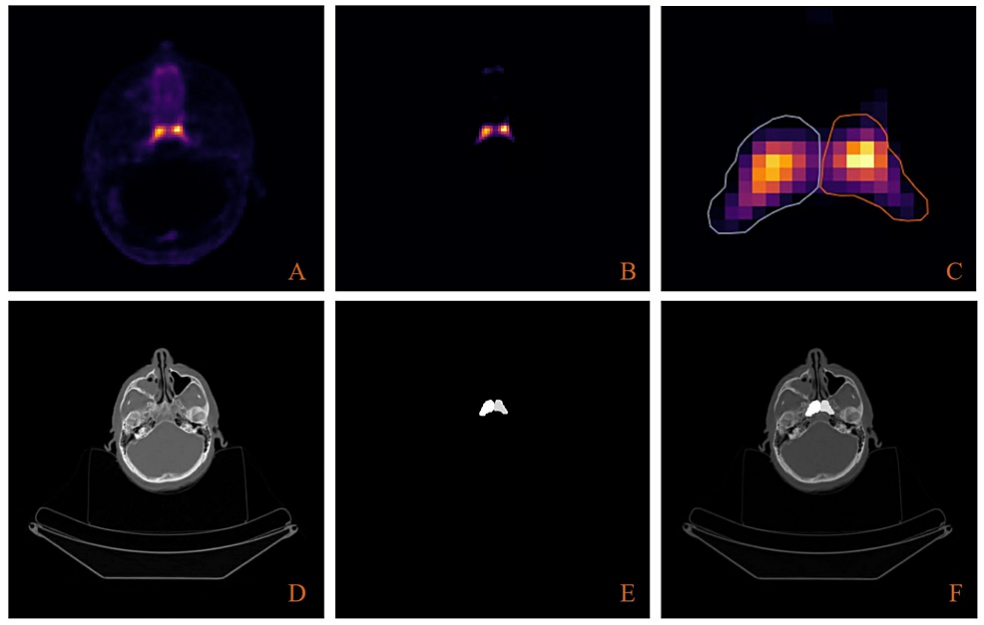
Overview of the manual tubarial gland delineation process. (A) PSMA PET images are converted to SUVbw. (B) A threshold is applied to images to illuminate tubarial gland perimeters. (C) Tubarial glands are contoured according to the threshold-defined perimeter. (D) CT and PET images are registered so that PET-derived contours and CT images use the same coordinate system. (E) Binary masks for each CT slice are created (1 inside the gland, 0 outside). (F) For visualization, the tubarial gland masks are shown overlayed with their corresponding location on the CT image (not used for training). PSMA: prostate-specific membrane antigen; PET: positron emission tomography; SUVbw: standard uptake value based on body weight.

An open-source program for the auto-segmentation of medical images, Organogenesis [19], was developed and used for pre-processing, model training, and post-processing of CT-predicted tubarial gland structures. Organogenesis is written in the Python programming language and its neural network models rely on the PyTorch package [20]. Data were preprocessed to create separate binary mask images for the right and left tubarial glands corresponding to each CT slice. CT images were normalized and PyTorch data loaders were initialized for iterating through the dataset during training. The process of using PSMA PET images to create tubarial gland masks for CT images is illustrated in Figure 1. Data were augmented by applying random elastic and perspective transformations included in the Albumentations Python library [21].

Model

The MultiResUNet architecture [22] was chosen for model development in this study. MultiResUNet is a deep learning architecture and was introduced in 2020 by Ibtehaz and Rahman as a potential successor to the U-Net architecture [8]. They made two major modifications to the U-Net architecture, one being the substitution of “MultiRes” blocks in place of double 3 x 3 convolutions. These blocks effectively enable the model to learn about image features at multiple resolutions by using convolutions of three kernel sizes in place of the one size used in U-Net. The other major innovation of MultiResUNet is the use of “Res paths” in place of skip connections. Whereas skip connections directly concatenate encoder and decoder outputs at multiple levels throughout the pipeline, Res paths first funnel encoder output through a series of convolutions and residual connections to avoid negative effects that may arise from concatenating high- and low-level features.

A two-dimensional (2D) MultiResUNet implementation was used, such that individual image slices are used as model input. Upon reviewing the literature to compare the performance of OAR segmentation models using 2D and three-dimensional (3D) U-Net [23,24], it was found that 2D U-Net often performs as well or better than 3D U-Net, and we, therefore, hypothesized that repeating model training with a 3D-architecture would not be significantly beneficial for the present study.

Model parameters

Models were trained for 20 epochs using a batch size of one and a learning rate of 0.001. These parameters were transferred from values obtained during prior experimentation with other HN OAR models in Organogenesis. A binary cross entropy loss function was used for gradient calculations and model parameters were updated using an Adam optimizer [25]. Models were trained using a 6 GB NVIDIA GeForce GTX 1060 (NVIDIA Corporation, Santa Clara, California) graphics processing unit (GPU).

Combined model for left and right tubarial glands

**Figure 2:**
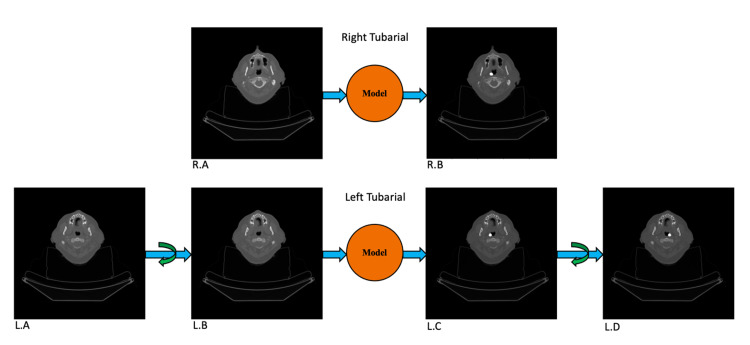
How a model for predicting tubarial glands on the right side of the body can be used for predicting left tubarial glands is illustrated. On the top row, the process for predicting a right tubarial gland is shown. In this case, a CT image slice (R.A) is input directly to the model, and a right tubarial predicted mask is generated (R.B). In this case, the mask is overlayed with the CT image for better visualization. On the bottom row, the process for predicting a left tubarial gland is shown. Before the original CT image (L.A) can be input into the model, it must first be flipped laterally (L.B). The mask generated by the model (L.C) must then be flipped laterally (L.D) to create the left tubarial gland mask image.

To maximize the training power of our limited available dataset, the bilateral symmetry of the tubarial glands was exploited to pool the right tubarial and left tubarial datasets into a combined dataset after first flipping the left tubarial CT images and masks about the patient’s posterior-anterior axis. This effectively doubles the size of the right tubarial dataset, and the combined dataset can then be used to train a single model for predicting tubarial glands. When predicting the right tubarial glands, CT images are fed straight into the model to obtain the right tubarial masks. When predicting left tubarial glands, CT images must be flipped about the posterior-anterior axis before being fed to the model. Similarly, the model output must be flipped about the posterior-anterior axis, to generate a left tubarial mask. This procedure is shown in Figure 2.

Model training/validation

To assess the generalized performance of the model [26], five-fold cross-validation was used for model training and evaluation. Five-fold cross-validation divides the dataset into five disjoint subsets, each containing six validation patients. For each validation set, the remaining 24 patients would become the respective training set. Within each of the five folds, separate models were trained with each training set and validated with the respective validation set. In this manner, five models were trained for both the left and right tubarial glands, which were all validated with independent validation sets. Model statistics were averaged over the five folds.

Post-processing

Model output is in the form of image masks for each CT slice, which are subsequently subjected to a sigmoid function, and then binarized via thresholding. The threshold value varies according to the predictive confidence and was determined iteratively for each model by minimizing the Dice similarity coefficient (DSC). Linear interpolation was used to fill in “gap” slices where no mask was predicted for an image while tubarial gland masks were predicted for image slices directly above and below the image. The Canny edge detection algorithm [27] was used to detect pixels containing tubarial edges in predicted mask images, which were then used to create tubarial contour arrays defined using the patient geometry from the CT metadata. Model-predicted tubarial gland contours were exported as DICOM radiotherapy structure set (RTSTRUCT) files.

Model assessment

To gauge model performance, the DSC, 95th percentile Hausdorff distance (HD95), and Jaccard similarity coefficient (JSC) were measured for using the validation set. The DSC quantifies the precision and recall of a model and when applied to Boolean data, is defined using the number of true positive (TP), false positive (FP), and false negative (FN) predictions.

$DSC = \frac{2TP}{2TP + FN + FP}$.

The Hausdorff distance is a maximin function, defined as the maximum distance from any point in one set to the nearest point in the other set (the 95th percentile of the distances is used to eliminate the impact of a very small subset of outliers). The JSC compares the similarity between two sets by taking the ratio of the intersection to the union.

$JSC(A,B) = \frac{A \cap B}{A \cup B}$.

The effective length and radius of predicted tubarial glands were calculated to compare manually delineated and auto-segmented gland sizes. The length was measured along the axial direction, and the gland radius was defined as the 90th percentile image slice radius, treating the contoured area from each image slice as a perfect circle.

$r = P_{90}\left( \sqrt{A/\pi}\right)$.

Differences between measurements of manually delineated and auto-segmented glands were assessed using a paired t-test.

## Results

**Figure 3:**
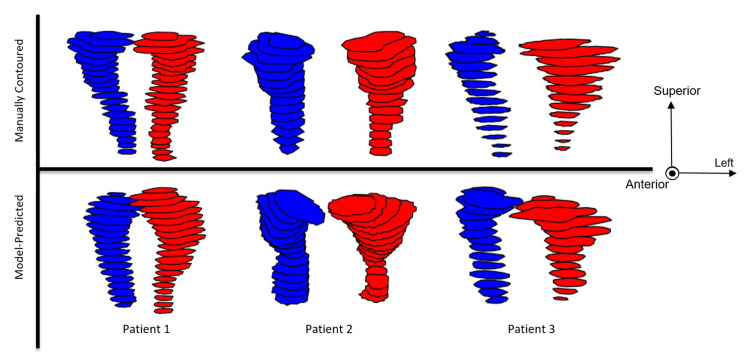
Manually delineated and auto-segmented tubarial glands are shown for three validation patients from a front-facing coronal perspective. The right glands are shown in blue and the left glands are shown in red.

**Figure 4:**
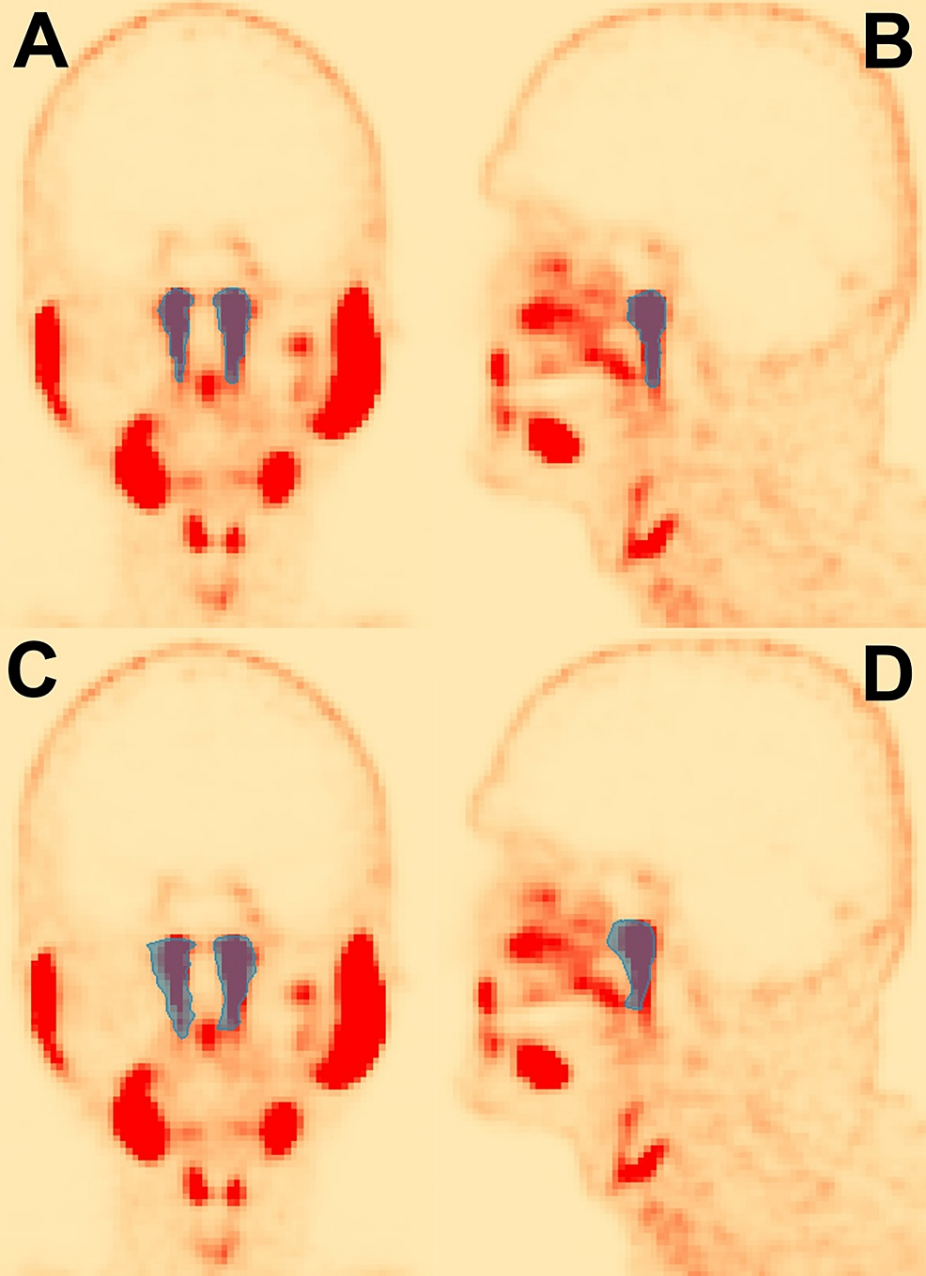
Manually delineated and auto-segmented tubarial glands are shown overlayed with PSMA PET images. (A) Coronal slice of manually delineated glands from the patient’s front. (B) Sagittal slice of manually delineated left tubarial gland from the patient’s left. (C) Coronal slice of auto-segmented glands from the patient’s front. (D) Sagittal slice of auto-segmented left tubarial gland from the patient’s left. PSMA: prostate-specific membrane antigen; PET: positron emission tomography.

Table 1 includes the DSC, HD95, and JSC statistics over the cross-validation. The tubarial gland model statistics averaged over the five-fold cross-validation were as follows: DSC of 0.64 ± 0.03, HD95 of 7.9 ± 2.0 mm, and JSC of 0.47 ± 0.03. Predicted contours qualitatively conform well to the general shape and size of manually delineated contours, as shown in Figure 3 with isolated contours and in Figure 4 with contours overlayed on PSMA PET images.

**Table 1:** Organogenesis models for the tubarial glands are assessed over a five-fold cross-validation with the Dice similarity coefficient (DSC), 95th percentile Hausdorff distance (HD95), and Jaccard similarity coefficient (JSC). Mean and standard deviations over the five folds are included.

Performance statistic	Cross-validation average
DSC	0.64 ± 0.03
HD95	7.9 ± 2.0 mm
JSC	0.48 ± 0.03

The length and radii of manually delineated and model-predicted tubarial glands are summarized in Table 2. The mean lengths calculated for manually delineated tubarial glands in this study were approximately 4.5 cm. Auto-segmented lengths were insignificantly (Table 2) larger than manually delineated lengths, with a mean length of 4.95 ± 0.98 cm. The average radius of manually delineated tubarial glands was 8.5 ± 1.1 mm, while the radius of auto-segmented glands was slightly larger at 9.3 ± 1.3 mm.

**Table 2:** Mean and standard deviation radius and length are shown for manually contoured and model-predicted tubarial glands (averaged over the five folds). The significance of the difference between statistics for manually delineated and model-predicted glands, calculated using a paired t-test, is included.

	Mean radius (mm)	Mean length (mm)
Manually delineated	8.5 ± 1.1	45.7 ± 8.5
Auto-segmented	9.3 ± 1.3	49.5 ± 9.8
Significance of difference	P = 0.013	P = 0.11

Using commodity hardware, auto-segmentation models created for the tubarial gland generated DICOM files in approximately one minute when utilizing an NVIDIA GeForce GTX 1060 (6 GB) GPU and 10 minutes when running strictly on an Intel Core i7-10700 (2.9 GHz; Intel Corporation, Santa Clara, California) central processing unit (CPU).

## Discussion

With ground-truth anatomical structures defined using PSMA PET images, a model for automatic segmentation of tubarial glands using only CT images has been developed. The current state of the model allows for an estimation of tubarial gland locations within CT images that can be used for HN radiotherapy plan optimization. Use of this model for tubarial gland delineation prior to radiotherapy requires low human effort and has the potential to positively impact patient outcomes when used to constrain dose to these regions, as it has been determined that dose levels to tubarial glands are a strong predictor of clinical toxicity [10]. The model and auto-segmentation program, Organogenesis, are available as open source, and the model can be improved upon by other institutions with access to further PSMA PET/CT data.

The DSC and HD95 between auto-segmented and manually delineated tubarial gland structures in this work are lower than values reported using state-of-the-art models for parotid glands [26]. It is important to remember that the tubarial gland models in this work were developed with a very limited dataset, and the aim of this work is not to present a model with conformity statistics competitive with those of state-of-the-art OAR auto-segmentation models trained with plentiful data, but rather to implement an initial CT-based auto-segmentation model for the tubarial glands, which can be iteratively improved upon, collaboratively. Furthermore, the cost of PSMA PET is prohibitive compared with CT, making it difficult to obtain large datasets for training. PET boundaries are also difficult to assess, making it difficult to create high-resolution tubarial gland contours.

While the current state of the model is inadequate for quantifying dose levels in the glands to a high degree of precision, due to its imperfect delineation of gland borders, it is still useful for approximating levels in the general region of the glands and could be effective for applying dose constraints to the general region of the tubarial glands. Optimistically, institutions that have access to PSMA PET data will be able to use the same technique used in this work to delineate the tubarial glands and continue training the existing model, improving its performance.

The tubarial glands were found to have high inter-patient variability in length and radius, and our dataset was not sufficiently large for our models to learn all predictive features on CT images to accurately characterize this variability. The variance observed is likely due to the use of SUVbw - defined tubarial gland borders, as SUVbw values are known to vary with patient mass [28]. The caudal (inferior) portion of manually delineated and auto-segmented glands was found to exhibit a narrow columnar shape, which was found to extend much further inferiorly in some patients than in others. The discrepancy in the lengths of auto-segmented and manually delineated tubarial glands may be partially explained by a model bias for delineating lower caudal regions.

Mean lengths calculated for manually delineated tubarial glands in this study were approximately 5 mm larger than previously observed values [10]. This is likely due to the choice of the threshold used for delineating the glands. Furthermore, the caudal region of the glands exhibits a thin, elongated, stem-like shape, which may reasonably not be fully included in their length calculation. Sample statistics of our small dataset may also deviate from population statistics.

In choosing the threshold for defining the interior of tubarial glands, we observed that the voxels in the HN region, which had a SUVbw greater than 1, were found within the regions of major salivary and tubarial glands, but only sparsely outside. Furthermore, SUVbw gradients along the perimeters of the glands were steep enough for small shifts in threshold choice to have little effect on gland delineation. These observations made SUVbw = 1 a suitable choice for the defining threshold for the tubarial glands.

The 90th percentile slice width was chosen to define the gland width as it is close to the maximum and more robust to noise associated with taking the maximum value from a distribution. Widths of manually delineated tubarial glands were slightly smaller than auto-segmented widths. This has statistical significance (Table 2), but the discrepancy is less than 1 mm, which is less than a single PET voxel and clinically insignificant.

Model performance was limited by the small cohort of 30 patients used for developing the models, which made it a challenge to decide the most effective way of splitting the data for training, validation, and testing. It is common for a certain percentage of data to be set aside as an independent training set that can be used for assessing model performance after training. This is most important if different predictive models are to be compared on the validation sets used for cross-validation. After consideration of allocating a small portion of our data to an independent test set, we decided the benefit of having a final set for assessing model performance would be outweighed by the decrease in size of our training set, as well as the questionable validity of such a small test set for assessing performance. A test set would have been required had we used our validation set to tune model architectures and hyperparameters, but for the purposes of this study, it was not a viable option.

Our model performance is also limited by our choice of model hyperparameters, which have not been fine-tuned using this dataset. It was not feasible for these parameters to be fine-tuned due to computational and time limitations. Our training rate was less than 15 epochs per day, resulting in over a week required to train five-fold cross-validation. Performing a nested cross-validation [26] to iterate over various hyperparameter permutations would require months of computation on our machine, which could not possibly be carried out for this study. Furthermore, fine-tuning of hyperparameters would be dependent on the validation set, which would require an external test set to be preserved for the final model assessment. Given our limited dataset, this was infeasible due to the negative impact on model performance that would result from shrinking the training set further.

Boundaries of tubarial glands cannot be seen on CT images, and the imaging space around the area of the glands has high inter-patient variability. While the model successfully learned the approximate region of the glands (learned from the training set patients), it is unable to predict glands with high conformity to the highly variable glands delineated with PSMA PET. Even with a larger training set, it will be a challenge for the model to learn CT image features that correlate with PSMA PET uptake in the tubarial glands, which is used to define ground truth image masks. To grasp the difficulty of this problem, imagine trying to train a model for segmenting dogs in photos where the lens cap has been accidentally left on the lens. Ground truth dogs are segmented on clear images of dogs taken during broad daylight, but then a model is created by training these masks to be predicted using featureless, “cap-on” images. In the present study, CT images (our “cap-on” images) do have features the model can work with, but not to the level of PSMA PET images.

Models for the segmentation of medical image structures are typically trained using much larger training sets, resulting in higher-level performance. While it may be argued that deep learning architectures are more appropriate for larger data sets, the use of deep learning is advantageous compared to other machine learning methods in this context due to the ability of deep learning model performance to scale with data. Although the dataset used for training this model was relatively small, a U-Net-based architecture was chosen such that the model can be incrementally improved upon as more data becomes available. The full Organogenesis program including the tubarial gland model is available such that groups having further PSMA-PET/CT datasets can continue training for improved model performance.

Dose in the tubarial glands has been correlated with post-treatment xerostomia [10]. The multicollinearity between parotid and tubarial gland dose for predicting xerostomia makes it difficult to predict an independent dose response of the tubarial glands, but it is possible that the lack of awareness around the presence of the tubarial glands has resulted in a missing variable, which could now be used to reduce the apparent uncertainty in salivary dose response. The dose response of the parotid glands has a high degree of inter-patient variability [29], and we suspect tubarial glands to share this variability. Given the similar PSMA-PET uptake of tubarial and parotid glands, as well as the previously demonstrated correlation between tubarial gland dose and xerostomia [10], it seems prudent to adopt parotid dose constraints for the tubarial glands until more specific guidelines become available.

The existence of tubarial glands is currently up for debate. Bikker and Vissink [14] argue that these glands should not yet be termed “salivary glands,” as they do not contribute to oral fluid in the oral cavity, and the histopathology suggests they are a collection of minor glands rather than single major glands. In a reply to comments, the original authors of the tubarial gland article [17] respond by noting that there is no evidence that the tubarial glands do not contribute to the oral fluid, that saliva is not strictly defined, and that all fluids contributing to the mixture in the oral cavity can be called saliva. Furthermore, they claim that the excretory duct openings of tubarial glands are macroscopically visible, and the major and minor gland classification system is limited. In another response to the tubarial gland article [17], it is argued that [16] there is no hard evidence for the existence of the tubarial glands, as neither PSMA PET/CT nor histology can prove their existence. The authors responded [17] by noting that they did not claim the existence of the glands based on either of these methods of discovery, but rather convey that the tubarial gland tissue has not previously been considered as salivary gland tissue but may need to be considered as an OAR for radiotherapy.

A clinical study to observe the effects of considering the tubarial glands as OARs during radiotherapy treatment planning could help resolve the lack of a definitive consensus on the role of these glands in radiotherapy. Our auto-segmentation models, freely available in Organogenesis, make this possible. Empirical evidence suggesting that dose to these glands plays an important role in patient salivary outcomes would be a convincing motivator for adopting tubarial glands as OARs.

## Conclusions

In conclusion, we have developed a CT-based auto-segmentation model for the newly discovered tubarial glands. PSMA-PET was used to delineate tubarial glands, which were then registered with corresponding CT images prior to training. The software is open source and available on GitHub including tubarial gland models, amongst auto-segmentation models for other OARs. Including tubarial glands in the optimization process of HN radiotherapy has the potential to improve patient outcomes with minimal effort, though further work is required to quantify this. Organogenesis can be used to quantify dose in the region of the tubarial glands for further evaluation and planning. The software for using this model is freely available and features cross-platform compatibility.
